# Modelling the short-term response to nitrogen that coordinates events in lateral root initiation

**DOI:** 10.1017/qpb.2026.10047

**Published:** 2026-05-05

**Authors:** Allison Gaudinier, Lisa Van den Broeck, Miguel Moreno-Risueno, Joel Rodriguez-Medina, Rosangela Sozzani, Siobhan M. Brady

**Affiliations:** 1 Plant and Microbial Biology, University of California Berkeley, USA; 2 Plant Biology and Genome Center, University of California Davis, USA; 3 Plant and Microbial Biology, NC State University, USA; 4 Centro de Biotecnología y Genómica de Plantas (CBGP), Universidad Politécnica de Madrid – Instituto Nacional de Investigación y Tecnología Agraria y Alimentaria, UPM-INIA/CSIC, Madrid, Spain; 5 Plant and Microbial Biology Department and NC Plant Sciences Initiative, North Carolina State University, Raleigh, NC, USA; 6 Howard Hughes Medical Institute, UC Davis, Davis CA95616, USA

**Keywords:** founder cells, lateral root initiation, mathematical modelling, nitrogen availability, transcriptional networks

## Abstract

Nitrogen (N) is an essential macronutrient and its bioavailability plays a major role in tuning plant development to the environmental nutrient status. To find novel factors in early root system architecture responses to N conditions, we performed *Arabidopsis thaliana* root transcriptome profiling of a short-term time course in limiting and sufficient N conditions. Using this data, we inferred transcriptional regulatory networks in each condition, which revealed the N-condition specific jasmonate responses. We found that the transcription factor (TF) ERF107 plays a generalized role in lateral root development while LBD13 is specific to N-limiting conditions. Further, we used a lateral root single-cell-identity specific transcriptome dataset to model the roles of TFs in early stages of lateral root development. We linked the N time course transcriptomics, lateral root mutant phenotypes and cell type-specific profiling approaches to find and test the roles of TFs that are involved in early root patterning responses to N conditions.

## Graphical abstract

**Figure:**
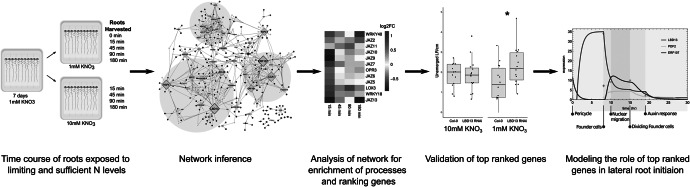

## 1. Introduction

Nitrogen (N) is an essential macronutrient for plants. However, bioavailable N levels are often insufficient, limiting plant growth and productivity. The sensing and uptake of N plays a crucial role in plant physiology and contributes to the regulation of many developmental and metabolic processes. As such, N is added via fertilizer to ensure sufficient plant growth and yield; however, this results in extensive downstream negative consequences to the environment (Wurtsbaugh et al., 2019). In the model species *Arabidopsis thaliana*, changes in N availability have a rapid impact on the transcriptome; many genes involved in N uptake and assimilation are upregulated within minutes of exposure to higher N concentrations (Krouk et al., 2010; Varala et al., 2018; Wang et al., 2007). These rapid responses allow for plants to take advantage of this potentially transient nutrient source. Additionally, N availability shapes root system architecture (RSA), largely by changes in lateral root initiation and elongation to scavenge N in a limiting environment (Gaudinier et al., 2018; Leyser & Day, 2009; Zhang & Forde, 2000). Changes in RSA are a common response to altered nutrient status in the environment, and allow the plant to tune its investment in root growth with its needs for nutrient acquisition (Leyser & Day, 2009). The factors that regulate these early transcriptional changes, which lead to N-responsive developmental changes in RSA, are largely unknown and those described do not sufficiently explain the N response.

Transcriptome datasets that profile expression in response to altered N conditions of ammonium and/or nitrate are diverse in their experimental design (different N concentrations and sources, and tissues sampled) and are collectively complementary as a data source to identifying genes that regulate N-mediated growth and metabolism. Temporal sampling of plants exposed to altered N conditions revealed rapid and dynamic expression changes for N transporters, metabolic genes and TFs (Krouk et al., 2010; Varala et al., 2018; Wang et al., 2007). These datasets, along with additional *in vitro* and *in vivo* data, can serve as input for network inference (Alvarez et al., 2020; Bargmann et al., 2013; Bian et al., 2025; Contreras-López et al., 2022; Gaudinier et al., 2018; Katari et al., 2010; O’Malley et al., 2016; Weirauch et al., 2014). Complex and interconnected transcriptional regulatory networks have been revealed (Vidal et al., 2020), including the identification of six transcription factors (TFs) as important regulators of N uptake and metabolism CIRCADIAN CLOCK ASSOCIATED 1 (CCA1), AUXIN RESPONSE FACTOR 8 (ARF8), BASIC LEUCINE-ZIPPER 1 (BZIP1), the TGACG SEQUENCE-SPECIFIC BINDING PROTEIN 1/4 (TGA1/4) double mutant, CYTOKININ RESPONSE FACTOR 4 (CRF4) and ABA RESPONSE ELEMENT BINDING/ABSCISIC ACID RESPONSIVE ELEMENT BINDING FACTOR (ABF)2 and ABF3 (Alvarez et al., 2014; Contreras-López et al., 2022; Gifford et al., 2008; Gutiérrez et al., 2008; Obertello et al., 2010; Varala et al., 2018). Furthermore, *bzip1*, *tga1/4, afb2* and *afb3* have RSA defects due to a misregulation of N metabolism, linking N-mediated changes in transcriptional regulation to root development. There is limited data, however, connecting short-term N exposure and its link with several aspects of lateral root development (Contreras-López et al., 2022; Varala et al., 2018). In this study, we addressed a knowledge gap through short-term transcriptional profiling of *Arabidopsis* roots in response to N concentrations that we previously described to have different effects on RSA (Gaudinier et al., 2018). Network inference and modelling were employed to uncover additional transcriptional regulators of N-responses that contribute to the developmental regulation of RSA. Our findings demonstrate that changes in N availability have a significant impact on transcriptional networks, notably those linked with lateral root development and jasmonate (JA) signalling. CCA1 was confirmed as a transcriptional regulator of N metabolism, while LOB DOMAIN-CONTAINING PROTEIN 13 (LBD13) emerged as a central regulator of N-mediated regulation, influencing lateral root emergence in a condition-specific manner. The interaction of LBD13 within a feedforward loop with PROTODERMAL FACTOR 2 (PDF2) and ETHYLENE RESPONSE FACTOR 107 (ERF107) was quantitatively modelled to simulate these regulatory interactions during early lateral root initiation. The model highlighted the importance of *LBD13’*s spatiotemporal expression for proper transcriptional progression in this critical mode by which roots alter their development during changes in N availability.

## 2. Results

### 2.1. Dynamics of nitrogen-responsive transcriptional networks show crosstalk with jasmonate signalling in Arabidopsis roots

**Figure 1.**
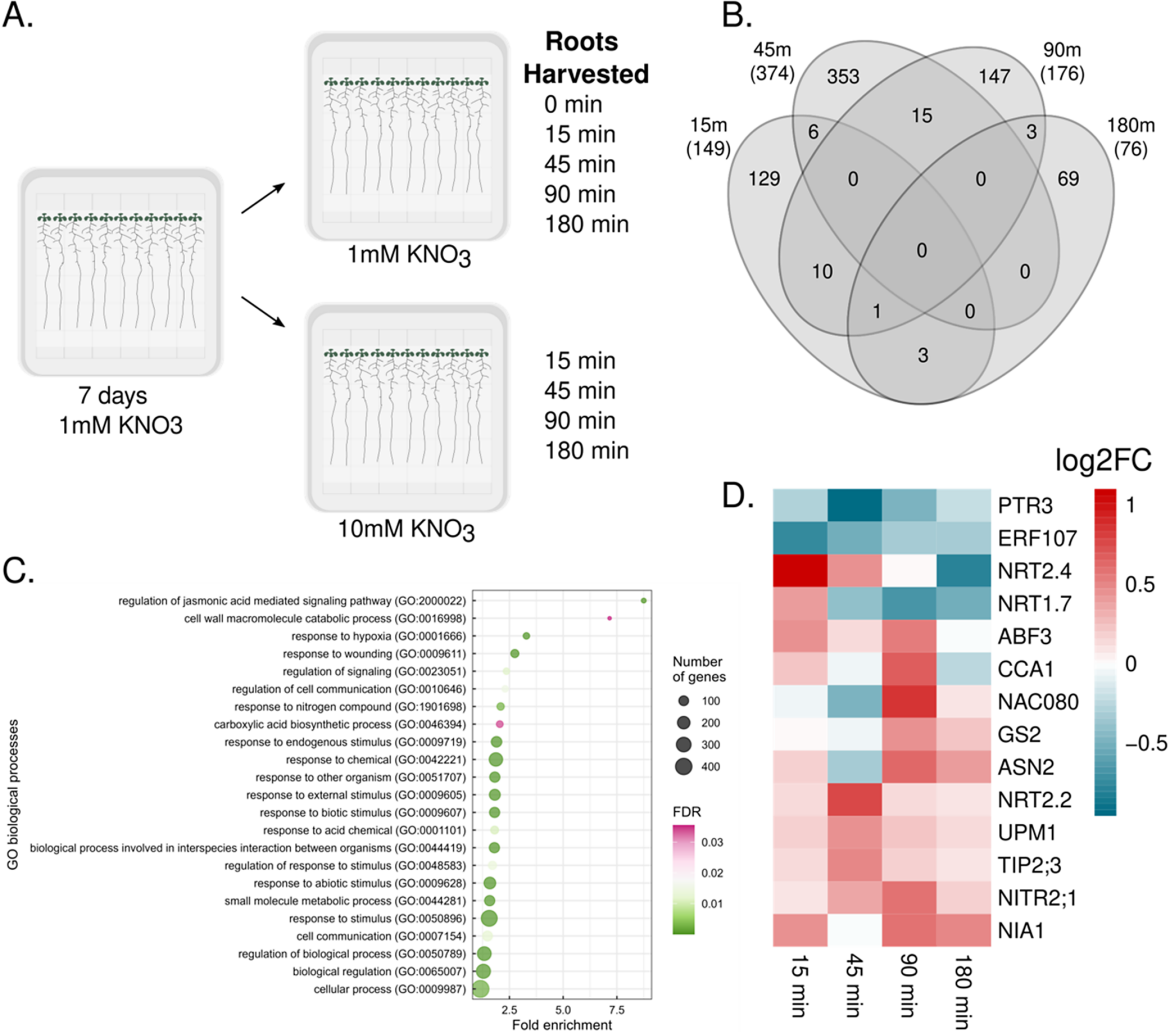
Transcriptional response upon nitrogen acclimation. (a) Experimental design of the time course. (b) Number of differentially expressed genes (DEGs) at each time point comparing nitrate sufficient with limiting conditions (*q* < 0.05). (c) Gene ontology enrichment of DEGs. (d) Heatmap of differentially expressed genes linked to N. Figure 1. long description.

As N is added to augment its limited bioavailability in the soil via fertilizer, we sought to characterize how a plant’s transcriptome changes upon a sudden increase in N concentration. *Arabidopsis thaliana* seedlings (Col-0) were grown for seven days on media with limiting nitrogen (1mM KNO_3_) and then transferred to either limiting (1mM) or sufficient (10mM) KNO_3_. Roots were collected immediately (0 minutes used exclusively for the 1mM KNO_3_ condition as a control for mechanical transfer), 15, 45, 90 and 180 minutes after transfer (Figure 1a). To identify transcriptionally responsive genes to this change in KNO_3_ concentration, we performed pairwise comparisons at each of these time points between N-sufficient and N-limiting conditions (q < 0.05). A total of 736 genes were detected as differentially expressed (DEGs), the majority of which showed distinctive and dynamic expression patterns. Specifically, 698 DEGs (94.84%) showed differential expression at only one time point (Figure 1b) and 38 DEGs (5.16%) were shared across at least two time points (Supplementary Figure 1, Supplementary Table 1). Such diversity in temporal transcriptional responses in response to a sudden increase in N suggests complex underlying regulatory networks.

To validate that our experimental design captured canonical aspects of the N response, we conducted enrichment analyses. When comparing our 736 DEGs against three diverse but complementary datasets that capture aspects of N-mediated transcriptional regulation, we found significant enrichment for N-responsive genes. Firstly, we found six DEGs in common with the fifty most broadly responsive N genes identified in a meta-analysis study (Canales et al., 2014) (Fisher’s exact *p*-value = 0.00211). In a comparable expression profiling dataset (Liu et al., 2017), we found 27/474 genes in common (Fisher’s exact *p*-value = 0.00017). Finally, we found an enrichment of our DEGs in our previously published yeast one-hybrid (Y1H) transcriptional network (Gaudinier et al., 2018) for N- and N-associated metabolism with 23/431 genes (Fisher’s exact *p*-value = 0.00126) (Supplementary Table 2A-C). Additionally, to further validate the robustness of our dataset, we performed a gene ontology (GO) enrichment and found that ‘response to nitrogen compound’ (GO:1901698) was significantly enriched within the DEGs (Figure 1c). When exploring the expression profiles for several nitrate transporters, nitrate assimilation genes and known nitrate-related transcription factors, we found that 11 of these genes showed a time point-specific induction, while 3 DEGs were repressed, highlighting the diverse temporal transcriptional nature of N-dependent responses (Figure 1d).

**Figure 2.**
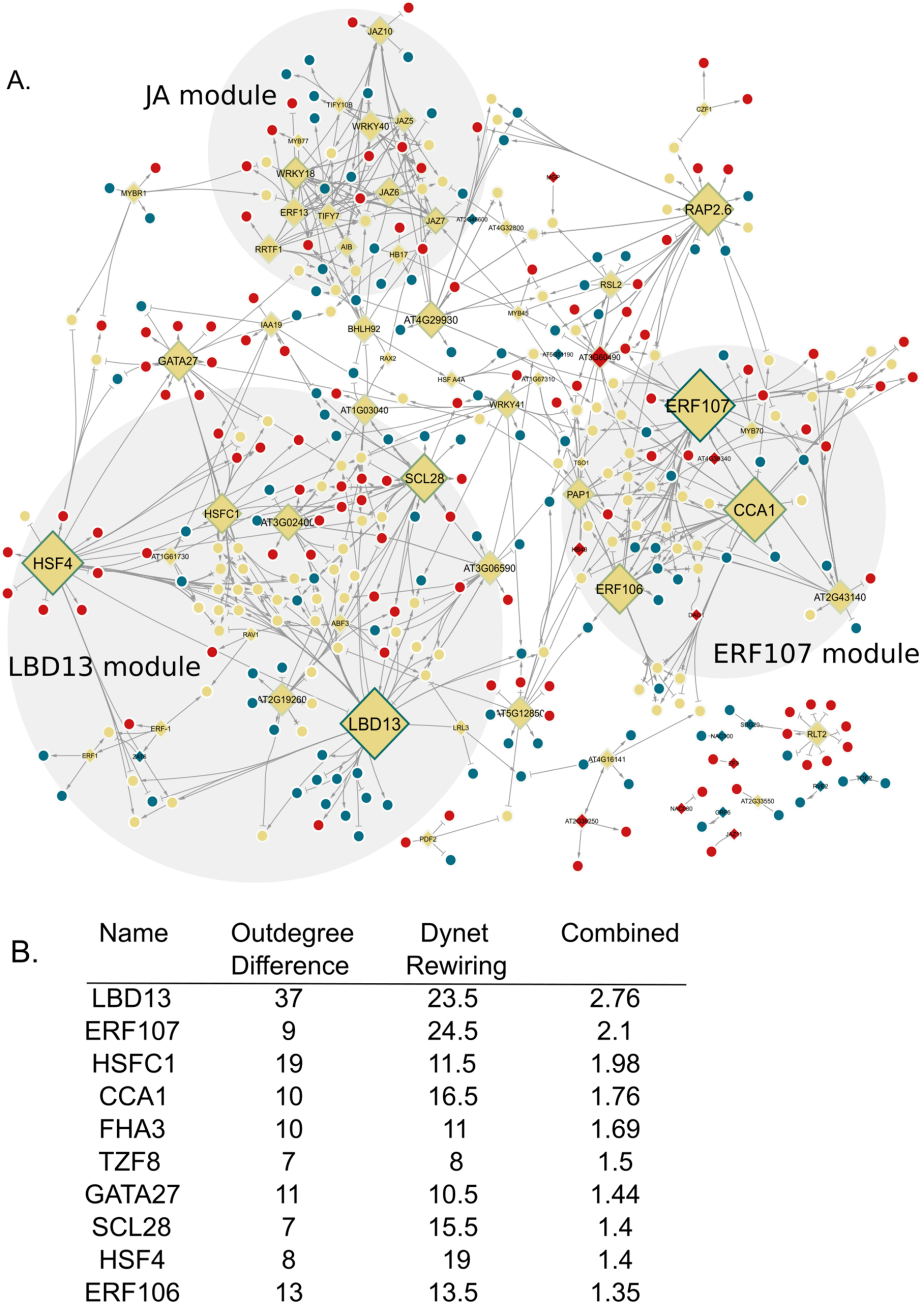
Network displaying the dynamic rewiring upon KNO_3_ exposure. (a) The border colour represents the DyNet rewiring score (darker is a higher score). Yellow nodes are genes in 1 mM KNO_3_ and 10 mM KNO_3_ networks, blue nodes are in the 1 mM network only, red nodes are in the 10 mM network only. Activating and repressing interactions are represented by point and block arrows, respectively. (b) Top 10 TFs from network analyses with outdegree difference and Dynet rewiring score combined into a value to rank importance in network structure. Figure 2. long description.

We then hypothesized that distinct transcriptional cascades result in a unique molecular response triggered at each time point upon differential nitrate availability. To identify transcriptional regulators enabling such a hierarchical response, we inferred gene regulatory networks (GRNs) with the previously identified 736 DEGs (FDR < 0.05), of which seventy-three are transcription factors (TFs) (Supplementary Table 3A). We inferred two networks using a Random Forest approach, one to explore regulatory interactions in nitrate-limiting conditions and the other in nitrate sufficiency. Changes in wiring (interactions between TFs and their downstream targets) were inferred using a machine learning approach (Spurney et al., 2020) (Supplementary Table 3B,C) (see Materials and Methods ). The network in N-limiting conditions contained 277 genes and 382 interactions, while the N-sufficient network comprised 279 genes and 384 interactions (Supplementary Figure 2A,B). In 28% of these inferred interactions, we found that the *cis*-elements present in the promoter of the target gene were bound by their respective TF *in vitro*, providing validation of our approaches (O’Malley et al., 2016) (Supplementary Table 4). When comparing these two networks (Figure 2a), major changes in connectivity were observed between JA signalling genes.

**Figure 3.**
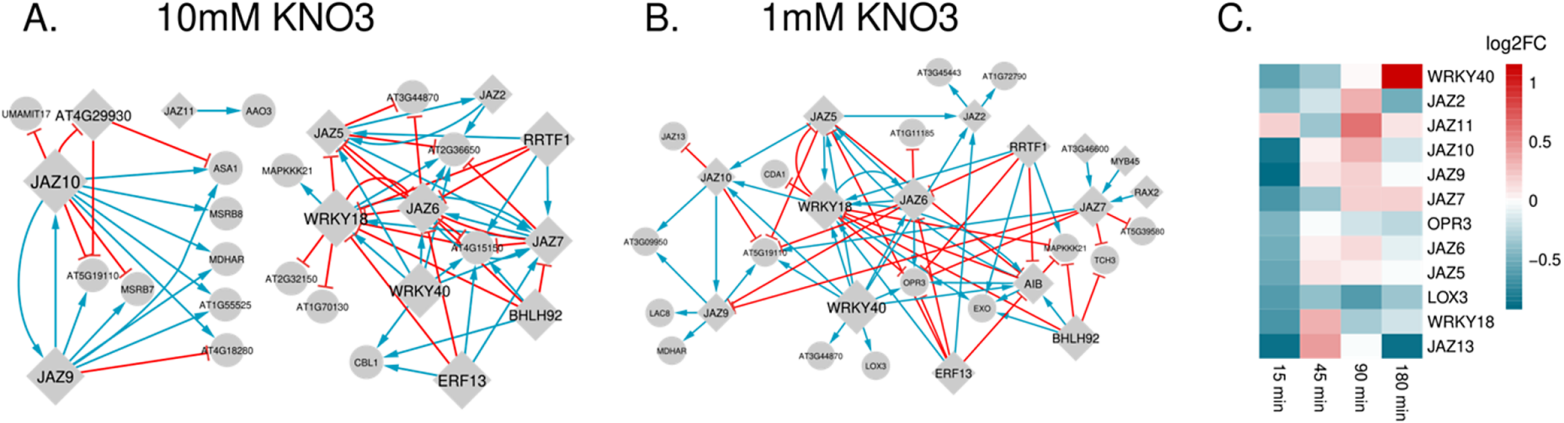
Rewiring of JA-related subnetwork upon KNO_3_ exposure. (a) 10 mM KNO_3_ network. (b) 1 mM KNO_3_ network. (c) Heatmap of differentially expressed genes linked to JA regulation and metabolism. Figure 3. long description.

Within this set of 736 DEGs used to infer the two networks, JA signalling (‘regulation of jasmonic acid mediated signalling pathway’ [GO:2000022]) was the most significantly enriched GO category (Figure 1c, Supplementary Figure 3 [heatmap for all JA genes]); and 171 of these genes were previously identified to be transcriptionally responsive to JA (Zhang et al., 2020) (Fisher’s exact *p*-value = 4.974e-13, Supplementary Table 2D). These genes include the JA precursor biosynthesis genes LIPOXYGENASE 3 (LOX3) (Bannenberg et al., 2009) and OXOPHYTODIENOATE-REDUCTASE 3 (OPR3) (Schaller et al., 2000), the JA catabolic enzyme JASMONATE-INDUCED OXYGENASE4 (JOX4) (Caarls et al., 2017), as well as JA signalling repressors, including eight JASMONATE-ZIM-DOMAIN PROTEIN (JAZ) proteins (Chini et al., 2007), and known JA-regulating TFs WRKY DNA-BINDING PROTEIN 18 (WRKY18) and WRKY40 (Pandey et al., 2010). Most of these JA signaling DEGs have reduced expression in sufficient nitrate, specifically at the 15-minute time point (Figure 3c, Supplementary Table 1). In addition to this over-representation, JA-associated genes form a highly interconnected module in both the limiting and sufficient nitrate inferred networks (Figure 2a). Despite their annotation as JA-associated genes, only 11 of the total 99 interactions across these modules are in common between the two networks (Figure 3a,b). Interactions with likely functional consequences include predicted targets of the JAZ transcriptional repressors (JAZ2, 5, 6, 7, 9, 13), as well as WRKY18 and 40, which are vastly rewired in the two N networks.

**Figure 4.**
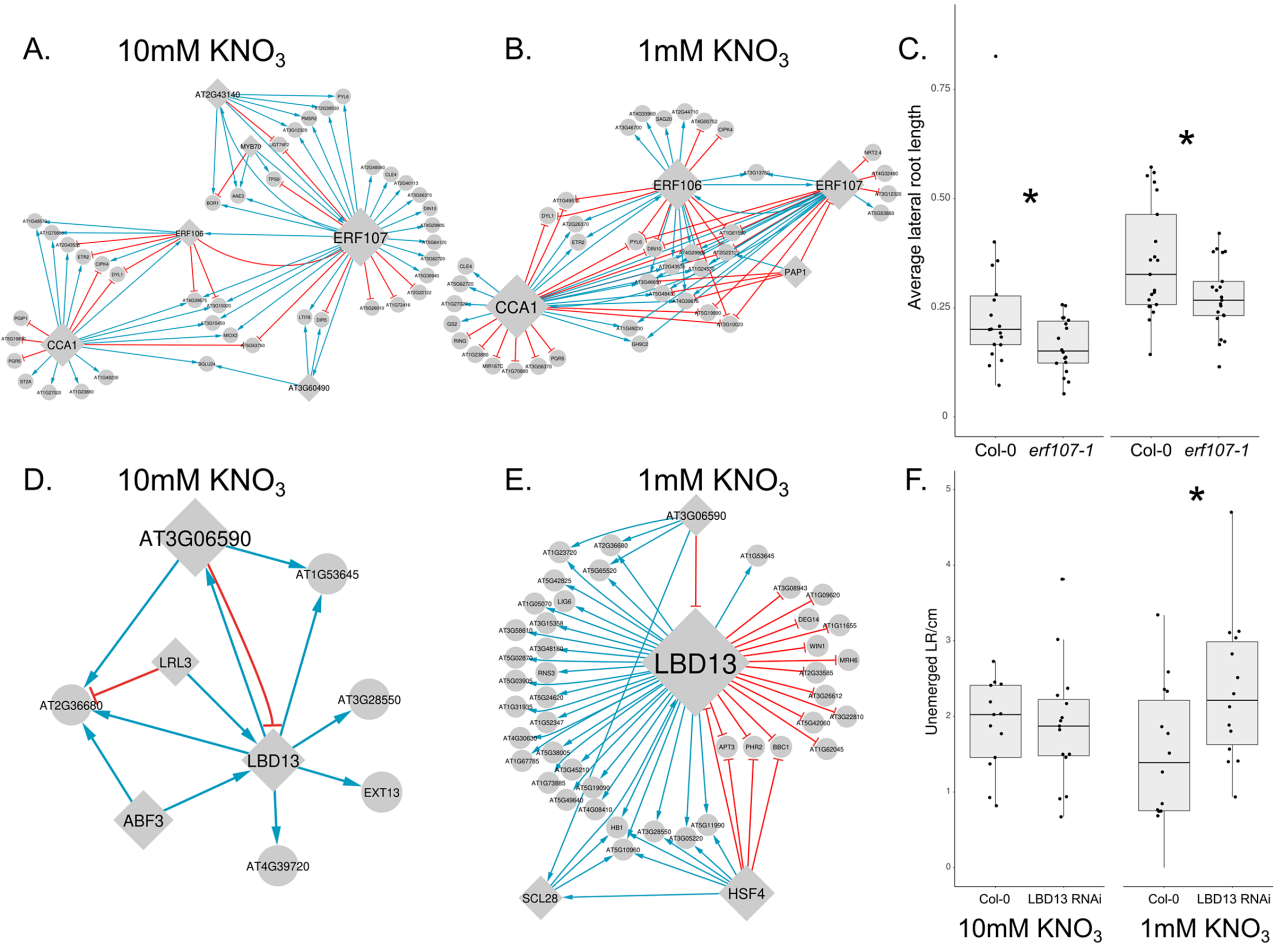
Rewiring of subnetwork linked to ERF107 and subnetwork linked to LBD13 upon KNO_3_ exposure. (a) ERF107 linked 10 mM KNO_3_ network. (b) ERF107 linked 1 mM KNO_3_ network. (c) *erf107-1* mutants have significantly shorter average lateral root lengths in 10 mM KNO_3_ and 1 mM KNO_3_. (d) LBD13 linked 10 mM KNO_3_ network. (e) LBD13 linked 1 mM KNO_3_ network. (f) LBD13 RNAi mutants have significantly more unemerged lateral roots per cm than Col-0 wild-type seedlings. ^*^Indicates *p*-value < 0.05 using two-way ANOVA with post-hoc Tukey HSD. Box plots are centred at the data median and mark from the 25th to the 75th percentile. Individual measurements are plotted as dots. For plot in c, *n* = 21 (1 mM KNO_3_), *n* = 18 (10 mM KNO_3_). For plot in f, *n* = 14 (1 mM KNO_3_), *n* = 13 (10 mM KNO_3_) Figure 4. long description.

### 2.2. Identification of key transcriptional players in the nitrogen-mediated network dynamics

Next, we delved into the identification of transcription factors (TFs) pivotal for the early transcriptional response to changes in N availability. Recognizing that TFs play a central role in the temporal rewiring of the network, we embarked on a comprehensive network analysis considering topology, regulatory rewiring and the impact of TFs on lateral root development upon perturbation (Brady et al., 2011; Jeong et al., 2001; Reich et al., 2001). To identify these key TFs, we ranked all the TFs taking into account network topology and rewiring. Specifically, we (1) quantified the outdegree of each TF, (2) computed the impact of each TF upon perturbation (Broeck et al., 2021), (3) used the DyNet plugin (Goenawan et al., 2016) in Cytoscape to summarize the regulatory rewiring of each TF between the two networks (Figure 2b). We ranked the TFs by normalizing each metric and, subsequently, taking their sum. Among the most highly ranked genes, we found CCA1, which has previously been shown to link the circadian clock with N metabolism (Gutiérrez et al., 2008). CCA1 also plays a role in root development: CCA1 overexpression results in altered lateral root architecture (Ruts et al., 2012). Within our network, we found that CCA1 participated in 30 interactions in the N-limiting network and 20 interactions in the N-sufficient network, with 13 of these interactions found in both networks. The eighth-ranked gene, SCARECROW-LIKE 28 (SCL28), participated in 13 interactions in the N-limited network and 20 interactions in the N-sufficient network, with only one of these interactions found in both networks. *SCL28* mutants have altered RSA with reduced primary root length (Goldy et al., 2021).

Further exploration into TFs crucial for the early transcriptional response led us to ERF106 and 107. Accordingly, genes that were differentially expressed between limiting and sufficient N conditions at 45 and 90 minutes after transfer formed a module generally clustered around the top-ranked CCA1, as well as ERF106 and ERF107. Under N limiting conditions, we inferred extensive overlap in direct downstream targets for ERF106, ERF107 and CCA1 (10 shared targets), ERF107 and CCA1 (5 shared targets), and between ERF106 and CCA1 (5 shared targets) (Figure 4a,b). This redundant regulation is less prominent in the N-sufficient network where we found 2 common targets with all 3 TFs, 4 between ERF107 and CCA1, and 6 between ERF106 and CCA1. Overall, the topology of this module, specifically the redundant regulation, may indicate the importance of this module in N limiting conditions. As such, we hypothesized that ERF106, ERF107 and CCA1 played an essential role in N-mediated regulation of RSA. To support this hypothesis, we further investigated ERF107. Inferred downstream targets of ERF107 were important N-related genes such as NRT2.4 and CCA1 in the N-limiting network. Indeed, we found that *erf107-1* mutants have reduced lateral root length in both limiting and sufficient nitrate (Figure 4c) (Gaudinier et al., 2018).

### 2.3. Deciphering LBD13’s role in nitrogen-mediated root development dynamics

LBD13 was the top-ranked gene in terms of the number of outgoing targets and its rewiring score (Figure 2b). Within the inferred network, LBD13 was part of a network module, which consisted of 51 genes (first-degree neighbours) that were primarily responsive to changes in N at the 180-minute time point. Notably, a majority of the regulatory interactions involving LBD13 (40/43) were specific to N-limiting conditions (Figure 4e). In N sufficient conditions, regulatory interactions for some of these genes are rewired with connections to other top genes: HEAT SHOCK FACTOR C1 (12 genes, 4 genes with the same interaction type), HEAT SHOCK FACTOR 4 (9 genes, 3 genes with the same interaction type) and SCL28 (1 gene with the same interaction type) (Figure 4d,e).

While LBD13 has previously been shown to regulate lateral root development, its function in response to changes in N availability is unknown. We used an inducible RNAi line of *LBD13* (Cho et al., 2019) and analysed its RSA in limiting and sufficient N in the presence of dexamethasone. In limiting N, we observed a differential lateral root response: the number of unemerged lateral roots per cm primary root was increased in N-limiting conditions relative to Col-0 control (Figure 4f). However, we observed no other RSA-related mutant phenotypes related to primary and lateral roots in the LBD13 RNAi line.

**Figure 5.**
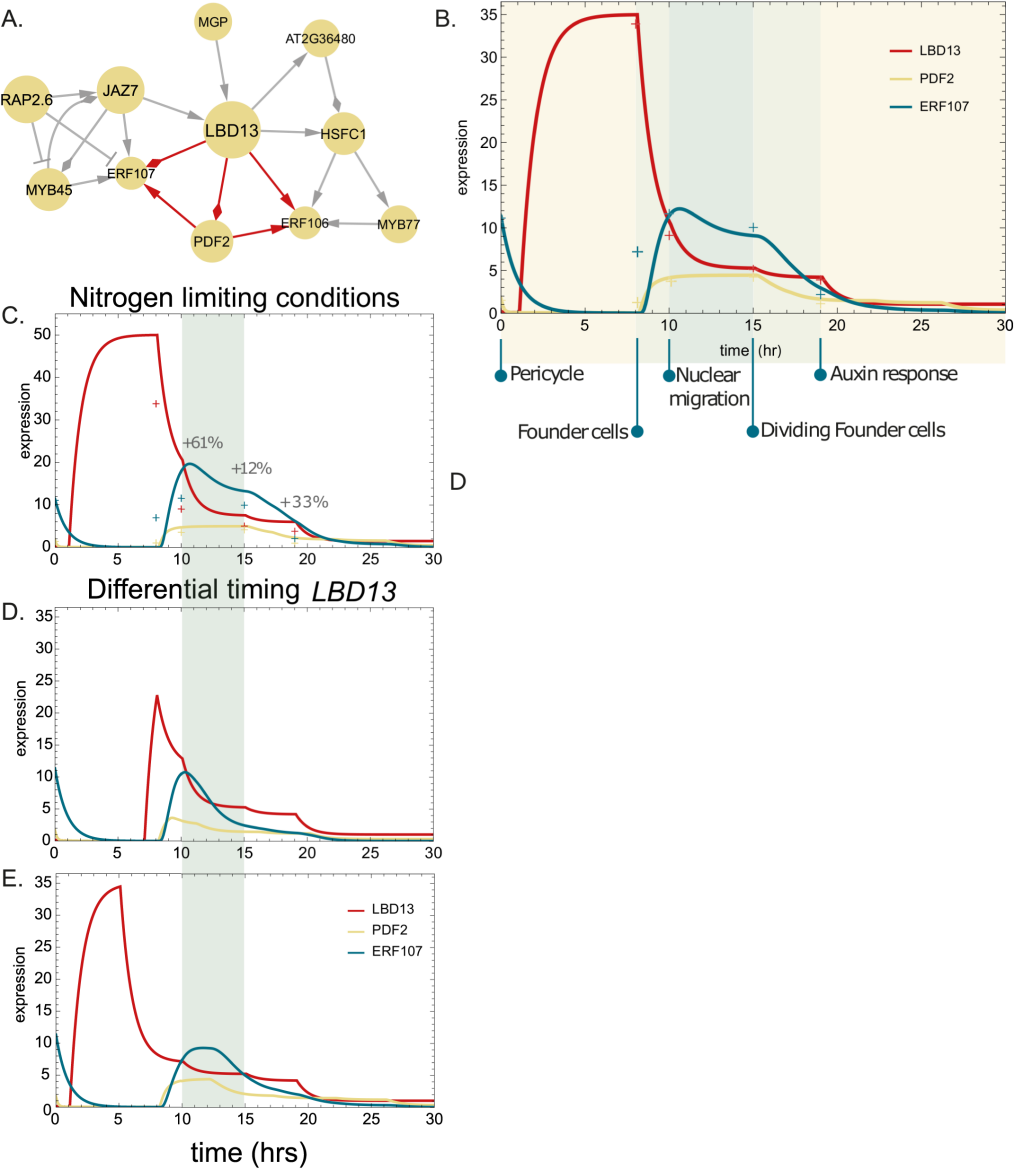
Mathematical modelling of the LBD13 feed-forward loop in lateral root development. (a) The regulatory interactions in which LBD13, PDF2, ERF106 and ERF107 are involved within the N-responsive TF regulatory network during lateral root development. (b–e) Expression of *LBD13*, *PDF2* and *ERF107* modelled in (b). A wild-type condition, (c) nitrogen-limiting conditions, (d,e) and differential timing of *LDB13* during lateral root development. Crosses on the graphs represent the experimental expression values at each development stage. Activating, repressing and undetermined interactions are represented by point, block and diamond arrows, respectively. Figure 5. long description.

Our transcriptomic time course data were obtained from RNA sequencing of bulk root samples. However, regulatory programmes comprising the initiation and elongation of lateral roots in different concentrations of available N are executed in a very small number of cells within the entire root. As the LBD13 RNAi line showed differential lateral root emergence in limiting vs sufficient N, we hypothesized that LBD13 may also play a role in the small number of cells that participate in early lateral root development. Indeed, *LBD13* had a peak of expression in lateral root founder cells, which declined as the founder cells progressed through asymmetric cell division and auxin response (Cabrera et al., 2024) (Supplementary Table 5). To further dissect the role of LBD13 in early lateral root development, we leveraged cell-type-specific single-cell expression profiling data that delineates a developmental trajectory associated with lateral root initiation in N-sufficient conditions and used dynamic Bayesian network inference to generate a TF regulatory network (TFRN) from the N-responsive TFs (Supplementary Table 3D, Supplementary Figure 5). Several biologically relevant network motifs were enriched within this early lateral root initiation network, including bi-fans, feed-forward loops (FFLs), feedback loops and bi-parallels (Milo et al., 2002) (Figure 5a, Supplementary Table 6). FFLs have been shown to be at the base of a plant’s adaptation to stimuli (Ma et al., 2009) and to play important roles throughout cellular development and growth, such as accelerating an output response and generating pulse-like dynamics (Joanito et al., 2018). We therefore further focused on a type I coherent feedforward loop (Mangan & Alon, 2003) in which LBD13 participates and for which there is pre-existing evidence of the target genes regulating early lateral root development. LBD13 is predicted to regulate PDF2 and ERF107; and PDF2 to regulate ERF107. LBD13 regulates the number of emerged lateral roots per cm of primary root (Figure 4f); ERF107 regulates lateral root length (Figure 4c) and PDF2 has been shown to function in lateral root development prior to emergence (Nagata & Abe, 2023; Nagata et al., 2021).

To analyse the expression dynamics of LBD13 and these two downstream targets in early lateral root initiation, and to link regulatory rewiring to a differential N response, we generated a quantitative model, using ordinary differential equations (ODEs); each of which included a production and degradation term that depended on its respective upstream regulators. We modelled LBD13 expression in a time-dependent manner based on cell-type expression data for single cell identities previously identified (Serrano-Ron et al., 2021). In addition, we included the homodimerization of PDF2 (Nagata & Abe, 2023), which led to a better fit of the model to the expression dynamics of its downstream target ERF107 as observed in the cell-type-specific single-cell expression data. When modelling a FFL, the two regulators, here LBD13 and PDF2, can act through an OR gate or an AND gate, where only LBD13 or PDF2 would need to be expressed to activate ERF107; or where both TFs need to be expressed to activate ERF107, respectively. The model fits the data better with an AND gate, suggesting that the expression of ERF107 is tightly regulated by multiple factors (Supplementary Figure 6). Moreover, our model suggests that the specific spatial expression of *LBD13* in the pericycle cells as they transition to founder cells is key for proper lateral root initiation (Figure 5b). The model demonstrated that LBD13 in the founder cells induces *PDF2* at the stage of nuclear migration and in dividing founder cells. Together, PDF2 and LBD13 activate *ERF107*, potentially inducing nuclear migration and founder cell divisions. To computationally predict how the dynamics among LBD13, PDF2 and ERF107 affect lateral root development upon nitrogen-limiting conditions, we adjusted *LBD13* expression in our model to its expression observed in our time course data under nitrogen-limiting conditions, which is a 43% increase. After running our model under these new conditions, we observed an increased and extended expression domain of PDF2 and ERF107 (Figure 5c).

We explored the robustness of our model by changing the duration of *LBD13* induction. Lower expression of *LBD13* at a later time point resulted in a lower peak of *ERF107* expression and no expression in the dividing founder cells (Figure 5d). In contrast, earlier expression of *LBD13* at the same magnitude as in the original model, but with a shorter duration, resulted in a peak of expression in founder cells during nuclear migration (Figure 5e). In each of these cases of modulation of *LBD13* expression magnitude and developmental stage, *PDF2* expression duration was the same (from nuclear migration to the initiation of the auxin response), but its relative magnitude shifted during these time periods (Figure 5c–e). The results show that *LBD13*’s spatiotemporal expression matters for the proper transcriptional progression of PDF2 and ERF107 and thus also potentially for lateral root initiation.

## 3. Discussion

In this study, we used a transcriptomic time course of roots exposed to limiting and sufficient nitrate conditions to infer an associated regulatory network. Here, we performed a time course experiment after N addition, profiling expression in roots using growth conditions we previously established to have significantly different effects on RSA (Gaudinier et al., 2018). While several time course experiments profiling aspects of the N response have been published (Krouk et al., 2010; Varala et al., 2018), experimental details such as N source and concentration, developmental age, organ type and time of day of harvest are all relevant factors when interpreting the resulting data. All these studies provide complementary datasets that can be mined to discover genes involved in the regulation of N-mediated growth and metabolism.

We analysed the RNA-seq time course data by identifying genes that were differentially expressed between the two N conditions at the same time point. The observed differential expression, therefore, is reflective of the altered N treatment as compared to our starting 0-minute time point. Taking these lists of DEGs and our knowledge of their temporal N responses, we used a machine learning approach with TuxNet to infer regulatory interactions and GRNs for each N condition. We used the network structure and regulatory modules as a guide to identify genes of interest. JA-responsive, signaling and biosynthesis genes are overrepresented within the network, and their importance is illustrated through its prominent size and its considerable rewiring between N conditions (Figure 2a). This suggests dynamic regulation of JA signalling in roots exposed to elevated N concentrations. JA pathway genes have been shown to affect lateral root growth (Cai et al., 2014; Hsu et al., 2013; Li et al., 2013; Raya-González et al., 2012; Wang et al., 2002) and are associated with the regulation of auxin production in roots, but there have been no direct connections made to link JA responses to N treatment to regulate RSA. These JA-related genes are initially upregulated, likely due to transfer of seedlings to new plates and are more highly expressed in the limiting N conditions (Supplementary Table 2D). This indicates that sufficient N in the environment may temper the JA-mediated plant mechanical stress response. Additional experimentation will be needed to confirm this transcriptional link between JA-mediated signalling and changes in N nutrient status within Arabidopsis roots.

Additional analysis of network connectivity and rewiring was performed to identify key TFs that demonstrated dynamic regulatory roles between the two networks. CCA1 and SCL28 were highly ranked and were previously demonstrated to play a role in root development (Goldy et al., 2021; Ruts et al., 2012). CCA1 and SCL28 have not been characterized in the context of altered N-dependent modulation of root system architecture. ERF106 and ERF107 clustered around the top-ranked CCA1 and comprise a module of interest, given their extensive target overlap and the N-related functions of these shared targets (Figure 4a,b). The prominence of ERF107 in both networks and the *erf107* mutant root phenotype (Figure 4c) support its role as a regulator of N-mediated root responses. Previously identified ERF107 target promoters, including prominent N uptake and biosynthesis genes such as NFP6.3/NRT1.1 and NIA1 (Gaudinier et al., 2018) were not identified in this inferred network. This may be due to spatio-temporal or N condition-specific aspects of the response that were not captured in our time course transcriptome. Collectively, these data demonstrate the success of our network analysis approach to identify likely critical TFs in this response. Nine of the top 10-ranked genes we identified (Supplementary Figure 4) have not been functionally assessed, and they deserve further experimental analyses for their role in N-mediated network temporal dynamics.

In accordance with the network ranking predictions, LBD13, our top-ranked TF, acts as an important regulator controlling lateral root emergence, specifically in limiting N conditions. This phenotype supports LBD13’s predicted role in the regulatory networks, where it is central to the structure of a module in the limited N network while having a minimal role in the sufficient N network. The network modelling approach applied to the time course transcriptome data and our subsequent analyses identified critical TFs and their putative targets. However, these bulk root transcriptome data will not facilitate the identification or analysis of gene targets that are expressed in the small number of cells that produce a lateral root. To overcome this dilution effect, we used a cell-type resolution lateral root transcriptome dataset and a distinct modelling approach that elaborated the LBD13 regulatory module, with two predicted targets, ERF107 and PDF2, that were found to act within a feedforward loop. Elucidation of the importance of these genes in the dynamic coordination of the lateral root response was conducted using ODEs coupled with a single-cell transcriptome dataset of very early steps in lateral root initiation. This modelling provides further evidence for the importance of LBD13 in orchestrating a FFL that plays an important role in the initiation of ERF107 expression and early events in lateral root initiation. This lateral root initiation is generally repressed; only when there are sufficient amounts of *LBD13* is initiation stimulated and the loop activated. In sufficient N conditions, *LBD13’*s expression is reduced and does not reach sufficient levels, leading to a reduced number of lateral roots. Taken together, our study identifies LBD13 as a central regulator in nitrogen-responsive transcriptional networks, finely modulating lateral root dynamics in response to specific N conditions. ERF106 was also involved in a feedforward loop, but with distinct temporal expression dynamics compared to ERF107. Further analysis of ERF106 mutants, modelling of this feedforward loop and its dynamics would extend our understanding as to the importance of these network motifs in determining root system architecture in the context of variable nitrate availability. Delving into these relationships will reveal emergent properties of the N networks, their structure and the possible redundancy that is a hallmark of many biological networks (Shen-Orr et al., 2002; Taylor-Teeples et al., 2015).

## 4. Materials and methods

### 4.1. Plant material and growth conditions

For sequencing libraries, Col-0 seeds were surface sterilized and plated on mesh on 1mM KNO_3_ media (recipe found in Gaudinier et al., 2018) and stratified for 2 days at 4°C. Plates were then placed vertically and grown at 22°C in 16 hour days/8 hour nights. Seven-day-old plants were transferred either to new 1mM or 10mM KNO_3_ plants at two hours post-dawn in the growth chamber. Per biological replicate, two plates per time point were transferred and then combined. Root tissue was then harvested and flash frozen in liquid N_2_ at 0 min (mesh was picked up and placed down on the same plate to account for mechanical responses to transfer), 15 min, 45 min, 90 min and 180 min. We performed four biological replicates for the time courses.

For RSA phenotyping, the LBD13 RNAi seeds were generously shared with us by Jungmook Kim (Cho et al., 2019). Col-0 and LBD13 RNAi seeds were surface sterilized and plated on 1mM KNO_3_ or 10mM KNO_3_ media containing 10 μM dexamethasone and were stratified for 2 days at 4°C. Plates were then placed vertically and grown at 22°C in 16 hour days/8 hour nights. Plants were grown for 7 days and imaged using a light box and a Canon EOS Rebel T7. Lateral root stages (emerged and unemerged) were quantified using a Nikon Diaphot TMD inverted microscope. Primary roots of 9-day-old seedlings were traced using a Wacom Bamboo tablet in ImageJ. Data were log-transformed and analysed using two-way ANOVA with post-hoc Tukey HSD in R.

### 4.2. RNA-seq library preparation and pooling of technical replicates

RNA-seq libraries were prepared following the BRAD-Seq DGE protocol (Townsley et al., 2015). Libraries were sequenced using the Illumina HiSeq 3000 in SR50 mode.

### 4.3. Transcriptome analysis and network inference

Reads of each sample were mapped against the *Arabidopsis thaliana* reference genome (TAIR v. 10) with TuxNet (Spurney et al., 2020) using default settings. TuxNet specifically uses ea-utils fastq-mcf for preprocessing (Aronesty, 2013), hisat2 to align the reads to the reference genome (Kim et al., 2015) and Cufflinks for differential expression analysis (Trapnell et al., 2012). To identify differentially expressed genes (DEGs), pairwise comparisons between both treatments at each time point were performed using an FDR < 0.05. Heatmaps and plots of the DEGs were generated in R (version 4.0.2) using ggplot2 (Wickham, 2016).

To infer gene regulatory networks, we first selected the short-term DEGs (i.e. DEGs at 15m, 45m, 90m and 180m) as the input set. Next, using the FPKM replicate values of the limited nitrogen dataset, we inferred a regulatory network between our input DEG set with a random forest approach (RTP-STAR) within the TuxNet interface. The regulatory interactions between the same set of DEGs were inferred within the sufficient nitrogen network by using the FPKM replicate values from the 10 mM KNO_3_ time course in the machine learning approach. Only putative TF-encoding genes were considered as source nodes that could regulate the expression of other DEGs. To infer a nitrogen-responsive network during lateral root development, the 73 nitrogen-responsive TFs were selected. Using Cabrera Chavez et al (Cabrera et al., 2024) cell-type specific expression dataset, we inferred a TF regulating network with Bayesian principles. Specifically, we used GENIST from the TuxNet interface. As settings, we used Reg Time Percent 0.5, Reg Fold Change Threshold 1.25 and Time Lapse 0 and 1. TuxNet is available at https://github.com/rspurney/TuxNet. Network visualizations were made using Cytoscape v3.8.0 (Shannon et al., 2003).

### 4.4. Network analyses

#### 4.4.1. Gene ontology enrichment

Enriched GO terms for genes within the inferred networks were identified with PANTHER. To summarize and reduce redundant GO terms, Revigo (Supek et al., 2011) was used. The GO enrichment plot was generated in R (version 4.2.2) using ggplot2 (Wickham, 2016).

#### 4.4.2. DNA affinity purification coupled with sequencing (DAPseq)

To test for additional support for our inferred regulatory interactions, we used previously published DAP-seq data (O’Malley et al., 2016). TF – DNA binding analysis data were downloaded from http://neomorph.salk.edu/dap_web/pages/index.php. Using a custom R script, we searched the database for TFs acting in our inferred networks and determined whether the promoter sequences of their target genes contained potential binding sites, based on the presence of cis-regulatory elements identified by DAP-seq experiments.

#### 4.4.3. N and JA enrichment

To test for enrichment of genes in the network using N-related datasets, Fisher’s exact test was used in R, using the standard function fisher.test(). For this, we used the gene list from Table 2 from Canales et al. (2014), Supplementary Table 2 from Liu et al. (2017) and Supplementary Table 3a from Gaudinier et al. (2018). We tested whether the overlap between differentially expressed genes within the inferred network was greater than that of differentially expressed genes that did not overlap with genes in the network.

To test for enrichment of genes in the network for jasmonic acid-related genes, we performed the same fisher.test() using the JA-responsive genes from Zhang et al. (2020),Supplementary Table S2).

### 4.5. Mathematical modelling

The mathematical model consisted of two ordinary differential equations (ODEs) for PDF2 and ERF107, and a time-dependent equation for LBD13. The interaction of PDF2, ERF107 and LBD13 was formulated as a type 1 coherent feed-forward loop with an AND gate. The spatial expression across lateral root development of LBD13 was modelled as time-dependent. The expression of PDF2 and ERF107 is under the control of LBD13 and LBD13 and PDF2, respectively. The regulatory interactions between these proteins were modelled using Hill equation dynamics. For the ODEs, it was assumed that transcription and translation happen quickly, such that transcription and protein degradation could be modelled in the same equation. All proteins are assumed to have a linear degradation term.

$$\begin{array}{r} \frac{dLBD13}{dt}=k_{1}LBD13 \end{array}$$

$$\begin{array}{r} \frac{dPDF2}{dt}=k_{2}\left(\frac{LBD13}{K_{D1}+LBD13}\right)-d_{2}PDF2 \end{array}$$

$$\begin{array}{rl} & \frac{dERF107}{dt}=\\ & \;k_{3}\left(\frac{LBD13\;PDF2^{2}}{K_{D1}PDF2^{2}+K_{D2}LBD13+LBD13\;PDF2^{2}+K_{D1}K_{D2}}\right)-d_{3}ERF107 \end{array}$$

OR gate

$$\begin{array}{rl} & \frac{dERF107}{dt}\\ & \;=k_{3}\left(\frac{K_{D1}PDF2^{2}+K_{D2}LBD13+LBD13\;PDF2^{2}}{K_{D1}PDF2^{2}+K_{D2}LBD13+LBD13\;PDF2^{2}+K_{D1}K_{D2}}\right)-d_{3}ERF107 \end{array}$$

Model simulation was done with Mathematica (Wolfram, Inc., Champaign, IN). The source code for the equations, model simulations and plotting are provided on https://github.com/LisaVdB/LBD13.

## Supporting information

10.1017/qpb.2026.10047.sm001 Gaudinier et al. supplementary material Gaudinier et al. supplementary material

## Data Availability

Transcriptomic data is available at https://www.ncbi.nlm.nih.gov/geo/query/acc.cgi?acc=GSE283555. Code is available on https://github.com/LisaVdB/LBD13.

## Supplementary material

The supplementary material for this article can be found at http://doi.org/10.1017/qpb.2026.10047.

## Figure 1. Long description

Panel A, top left, is a flowchart showing seedlings grown for 7 days on 1 m M K N O sub 3, then transferred to either 1 m M or 10 m M K N O sub 3. Roots are harvested at 0, 15, 45, 90, and 180 minutes for 1 m M, and at 15, 45, 90, and 180 minutes for 10 m M. Panel B, top right, is a Venn diagram with four overlapping ovals labeled 15 m, 45 m, 90 m, and 180 m, each with numbers indicating the count of differentially expressed genes at each time point and their intersections. For example, 353 genes are unique to 45 m, 149 to 15 m, 147 to 90 m, and 76 to 180 m, with smaller numbers in overlapping regions. Panel C, bottom left, is a dot plot with Y axis labeled G O biological processes and X axis labeled Fold enrichment. Dots represent enriched processes among differentially expressed genes, sized by number of genes and colored by F D R. Top processes include regulation of jasmonic acid mediated signaling pathway and cell wall macromolecule catabolic process. Panel D, bottom right, is a heatmap with genes listed on the Y axis (P T R 3, E R F 107, N R T 2.4, N R T 1.7, A B F 3, C C A 1, N A C 080, G S 2, A S N 2, N R T 2.2, U P M 1, T I P 2;3, N I T R 2;1, N I A 1) and time points on the X axis (15 min, 45 min, 90 min, 180 min). Color scale represents log 2 fold change, with red for upregulation and blue for downregulation.

Navigate back to Figure 1..

## Figure 2. Long description

Panel A is a network diagram with nodes and directional edges. Three main modules are shaded: L B D 13 module at lower left, J A module at upper center, and E R F 107 module at lower right. Nodes are colored yellow if present in both 1 milli molar and 10 milli molar K N O 3 networks, blue if only in 1 milli molar, and red if only in 10 milli molar. Node borders vary in darkness, with darker borders indicating higher DyNet rewiring scores. Large diamond-shaped nodes labeled L B D 13, H S F 4, S C L 28, R A P 2.6, E R F 107, and C C A 1 are prominent. Arrows with points represent activating interactions, while block arrows indicate repressing interactions. Panel B is a table listing the top ten transcription factors: L B D 13, E R F 107, H S F C 1, C C A 1, F H A 3, T Z F 8, G A T A 27, S C L 28, H S F 4, and E R F 106. Columns are Outdegree Difference, DyNet Rewiring, and Combined, with L B D 13 scoring highest in all categories. The table is read left to right, with values: L B D 13 (37, 23.5, 2.76), E R F 107 (9, 24.5, 2.1), H S F C 1 (19, 11.5, 1.98), C C A 1 (10, 16.5, 1.76), F H A 3 (10, 11, 1.69), T Z F 8 (7, 8, 1.5), G A T A 27 (11, 10.5, 1.44), S C L 28 (8, 15.5, 1.4), H S F 4 (8, 19, 1.4), E R F 106 (13, 13.5, 1.35).

Navigate back to Figure 2..

## Figure 3. Long description

Panel A on the left shows a network diagram labeled 10 m M K N O sub 3, with circular and diamond-shaped nodes such as J A Z 10, J A Z 9, W R K Y 40, and E R F 13. Blue and red arrows connect nodes, indicating regulatory relationships. Panel B in the center is a similar network for 1 m M K N O sub 3, with more densely connected nodes including J A Z 10, J A Z 9, J A Z 7, W R K Y 40, and O P R 3. The pattern and density of arrows differ from panel A, with more red arrows. Panel C on the right is a heatmap labeled log 2 F C, with gene names listed vertically: W R K Y 40, J A Z 2, J A Z 11, J A Z 10, J A Z 9, J A Z 7, O P R 3, J A Z 6, L O X 3, W R K Y 18, J A Z 13. Time points are listed horizontally: 15 min, 45 min, 90 min, 180 min. Color intensity ranges from red (upregulation) to blue (downregulation), showing dynamic expression changes for each gene across time points.

Navigate back to Figure 3..

## Figure 4. Long description

Panel A at top left shows a network diagram for 10 mM K N O sub 3 with ERF107 as a central node, connected by blue and red edges to multiple genes, and CCA1 as a secondary hub. Panel B at top center shows the 1 mM K N O sub 3 network with ERF107, ERF106, and CCA1 as central nodes, with altered edge patterns compared to panel A. Panel C at top right is a box plot with x-axis labeled Col-0 and erf107-1 for both 10 mM and 1 mM K N O sub 3, y-axis labeled average lateral root length, showing significantly shorter roots in erf107-1 mutants, indicated by asterisks. Panel D at bottom left shows a network for 10 mM K N O sub 3 with LBD13 as the central node, connected to AT3G06590, LRL3, ABF3, EXT13, and others, with directional arrows. Panel E at bottom center shows the 1 mM K N O sub 3 network with LBD13 as the main hub, connected to more genes than in panel D, including HSF4. Panel F at bottom right is a box plot with x-axis labeled Col-0 and LBD13 RNAi for both 10 mM and 1 mM K N O sub 3, y-axis labeled unemerged L R per cm, showing significantly more unemerged lateral roots in LBD13 RNAi mutants, marked by asterisks. All box plots display medians, interquartile ranges, and individual data points.

Navigate back to Figure 4..

## Figure 5. Long description

Panel A at the top left is a network diagram with L B D 13 at the center, connected by arrows to P D F 2, E R F 106, and E R F 107. Red arrows indicate activating, block arrows indicate repressing, and diamond arrows indicate undetermined interactions. Other nodes include R A P 2.6, J A Z 7, M Y B 45, M G P, A T 2 G 36480, H S F C 1, and M Y B 77, connected by gray lines. Panel B at the top right is a line graph with x-axis labeled time (hr) from 0 to 30 and y-axis labeled expression from 0 to 35. Three lines represent L B D 13 (red), P D F 2 (orange), and E R F 107 (blue). Key developmental stages are marked below the x-axis: Pericycle, Founder cells, Nuclear migration, Dividing Founder cells, and Auxin response. L B D 13 peaks early, P D F 2 and E R F 107 peak later. Panel C below B is labeled Nitrogen limiting conditions, with the same axes and color scheme. L B D 13 expression drops sharply after peaking, P D F 2 and E R F 107 show lower, delayed peaks. Percentage changes (+61%, +12%, +33%) are annotated. Panel D below C is labeled Differential timing L B D 13, showing a shifted peak for L B D 13 and corresponding changes in P D F 2 and E R F 107. Panel E at the bottom shows all three lines with similar axes and color scheme, summarizing expression dynamics over time. Crosses on the graphs indicate experimental data points at each developmental stage.

Navigate back to Figure 5..
