# Supplementary figures and images for: Modelling the short-term response to nitrogen that coordinates events in lateral root initiation
Source: Quant Plant Biol. 2026 May 5;7:e12. doi: 10.1017/qpb.2026.10047 (PMC13280857; doi:10.1017/qpb.2026.10047)

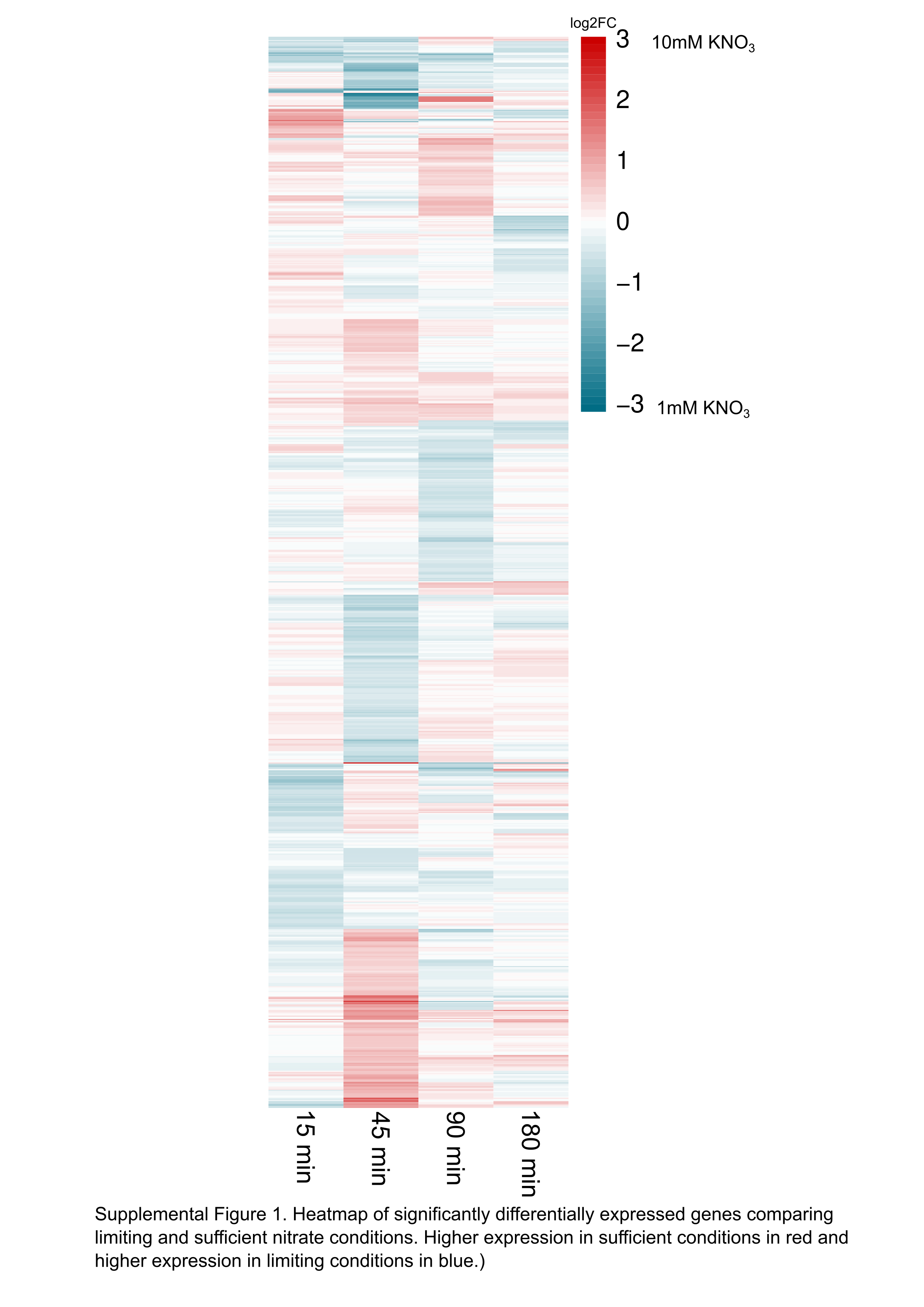

Supplement: Gaudinier et al. supplementary material [file S2632882826100472sup001.zip › FigureS1.tiff]

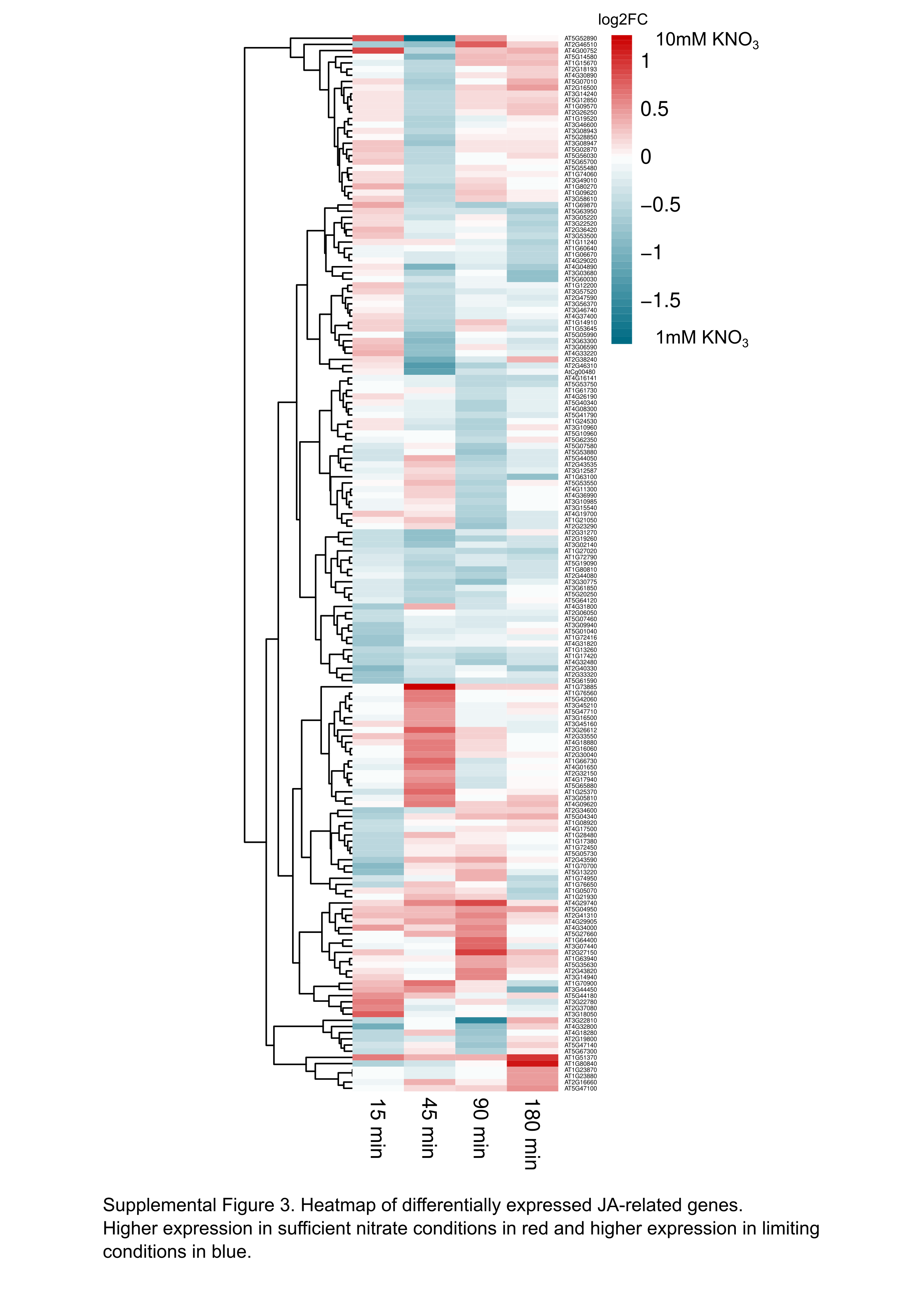

Supplement: Gaudinier et al. supplementary material [file S2632882826100472sup001.zip › FigureS3.tiff]

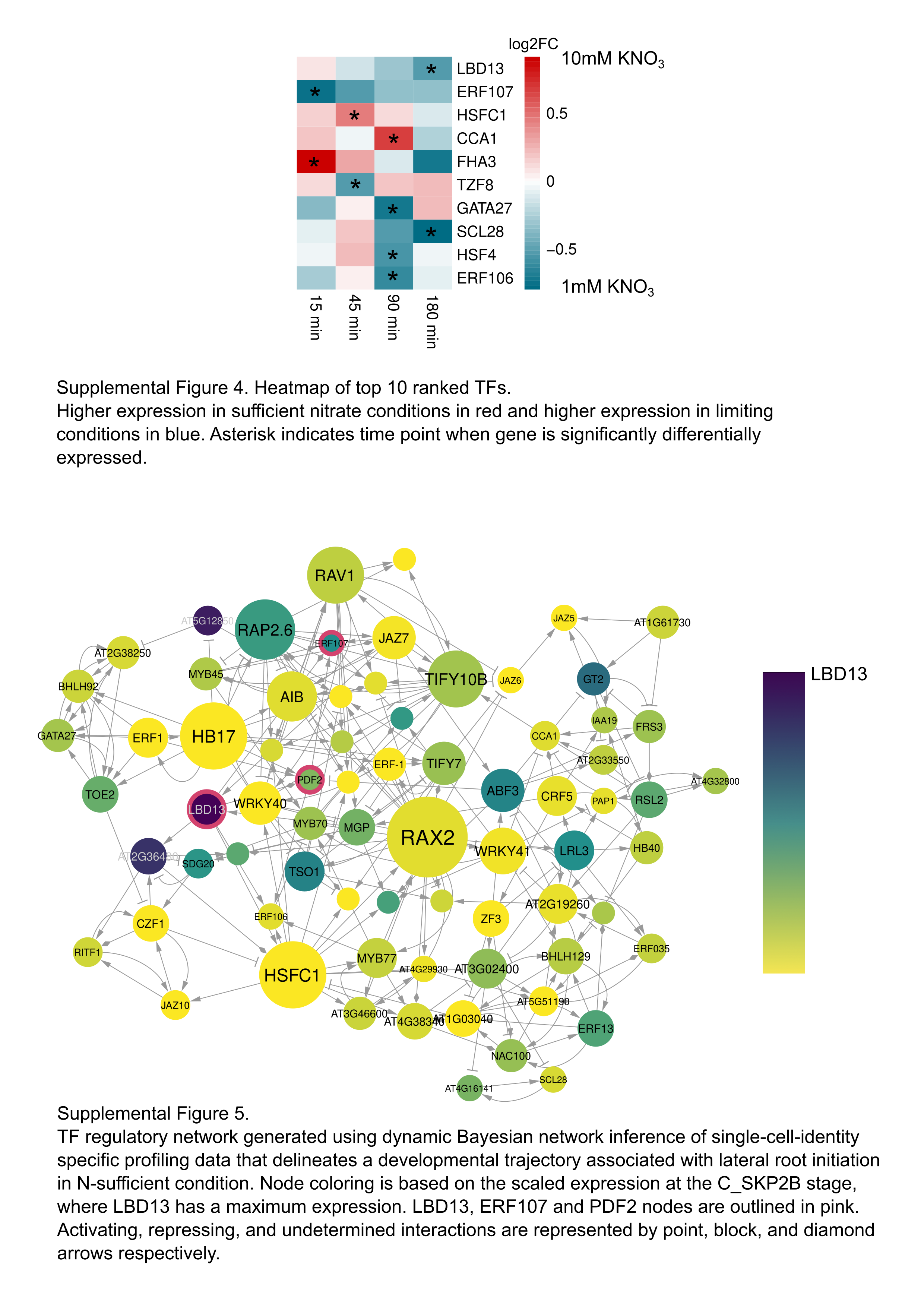

Supplement: Gaudinier et al. supplementary material [file S2632882826100472sup001.zip › FigureS4&5.tiff]

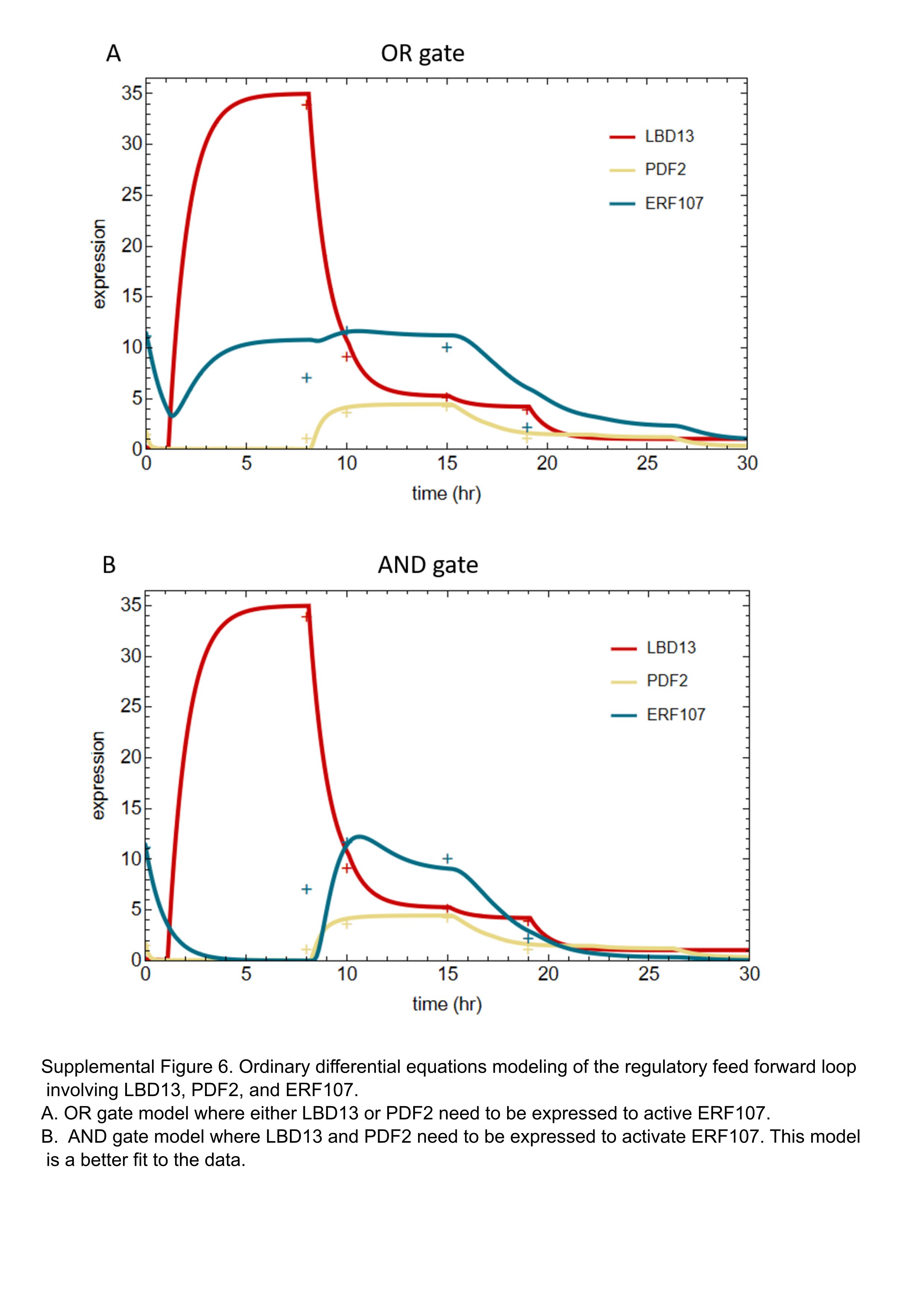

Supplement: Gaudinier et al. supplementary material [file S2632882826100472sup001.zip › FigureS6.tiff]
