# Supplementary material for: Modelling the short-term response to nitrogen that coordinates events in lateral root initiation
Source: Quant Plant Biol. 2026 May 5;7:e12. doi: 10.1017/qpb.2026.10047 (PMC13280857; doi:10.1017/qpb.2026.10047)
Supplement: Peer review history

# Reviewer report: Review: Modelling the short-term response to nitrogen that coordinates events in lateral root initiation — R0/PR2

**DOI:** 10.1017/qpb.2026.10047.pr2

The manuscript QPB-2025-0014 entitled “Modeling the Short-Term Response to Nitrogen that Coordinates Events in Lateral Root Initiation” focused on discovering novel genes related to the early root system architecture responses to N conditions in Arabidopsis.

By using a root transcriptome comparison of a short-term time course between optimum and limiting N conditions. More interestingly, the authors used a single-cell specific transcriptome dataset to model the roles of TFs in early stages of lateral root development.

The manuscript furnishes evidence on the roles of specific TFs that are involved in early root patterning responses to N conditions and these findings are really interesting for improving the knowledge about the role of some genes in modifying RSA to cope with N limited conditions.

Therefore, I found some major and minor constraints in the structure of the manuscript and some missing information in MM section, in detail the major:

1. The Authors exchange the results section with a discussion, based on the addition of several citations in the results that in general could be only the presentation of own results. As a consequence, the discussion section resulted poor of details that probably are already discussed in the results. Thus, I warmly suggest the Authors to restructuring both results and discussion sections in agreement with my suggestion.

2. MM section missed some information and it is organized putting inside different activities (e.g. why DAP seq is included in the subsection “Network Analyses”?) generating a lot of confusion. Some subsections, as DAP seq, should be presented with many additional information.

Minor comments:

1. In some section there are some citations by the number together with the authors and year of publication, please verify

2. The quality of some figures could be improved (e.g. Figure 1A and 1B)

3. Despite many Authors are native language, the English style should be improved in several sections, starting from the abstract

4. I suggest the Authors to modify the manuscript title including “N limiting” and “TFs” that coordinates events in “lateral root formation”

# Reviewer report: Review: Modelling the short-term response to nitrogen that coordinates events in lateral root initiation — R0/PR3

**DOI:** 10.1017/qpb.2026.10047.pr3

**Authors:** Thomas Blein

**Affiliations:** Universite Paris Saclay

In this article, Gaudinier et al. investigate the short-term root transcriptional response of Arabidopsis thaliana to changes in nitrogen (N) availability to identify key factors and regulations involved in lateral root initiation. First, the authors conduct a transcriptomics time series from low to high N concentration, including the corresponding unchanged controls, focusing on the early time points (15 min, 45 min, 90 min, 180 min). They show that among the 736 differentially expressed genes (DEGs), the majority is only differentially expressed at a single time point. The DEGs are enriched in known N-responsive genes, in agreement with previous comparable analyses. Based on this time series, the authors reconstruct two gene regulatory networks (GRNs): one from the N-deficient condition and one from the N-sufficient recovery condition. By comparing the two GRNs, the authors identify a module enriched in JA signaling genes as being largely rewired between the two GRNs. Then, the authors search for the key transcription factors of the GRN based on the out-degree, the potential TF impact on GRN, and the rewiring occurring between the two GRNs. Several of these TFs have been shown to have root architecture defects when modified in the literature (CCA1 and SCL28) or based on experiments done in this paper (ERF107 and LBD13), and the authors notably link together CCA1, ERF106, and ERF107. LBD13 was the TF with the strongest rewiring score; the majority of regulations were specific to the N-deficiency condition and regulate mainly DEGs at the 180 min time point. LBD13 downregulation showed a significant lateral root emergence phenotype, suggesting its impact on early LR development. Therefore, the authors reconstruct a GRN with the N-responsive TFs based on single-cell data of LR development previously generated. Notably, they identify that LBD13 and PDF2 regulate ERF107 in a feed-forward loop. They model this regulation through ordinary differential equations and confirm the regulation and timing observed in single-cell data, noting that both LBD13 and PDF2 are required for the induction of ERF107. Using this model, they show that in N-deficiency conditions, the expression of PDF2 and ERF107 is higher and more extended in time, and that LBD13 expression timing is key for the correct expression duration of both PDF2 and ERF107 genes.

# Major comments

1) In this article, the authors identify three interesting regulation modules: 1) JA-responsive genes; 2) the CCA1/ERF106/ERF107 regulation module; and 3) the LBD13/PDF2/ERF107 module. However, the investigations are a bit disconnected. JA could indeed be linked to the transfer required by the experiment design, but the LBD13/PDF2/ERF107 regulation module does not seem to appear in the time-course GRNs. What about the other regulations inferred by the single-cell GRN? A more in-depth comparison of the different networks would be valuable for the paper and would better link the different parts.

2) Using the LBD13 RNAi line, you should be able to confirm the regulations inferred by modeling, notably those of PDF2, ERF106, and ERF107.

3) ERF106 is also involved in a feed-forward loop involving LBD13 and PDF2. Using a comparable ODE model to the one used for ERF107, are the regulations of ERF106 the same as those of ERF107?

# Minor additional comments

Figure S2: A color legend for the time points is missing.

Figure 5A: The description of the different shapes of arrowheads is missing from the legend (it is only present in Figure S5). Please add it to Figure 5A as well.

Figure 5C: Are you sure that this represents the N-sufficient condition and not the N-deficient condition?

# Editor report: Recommendation: Modelling the short-term response to nitrogen that coordinates events in lateral root initiation — R0/PR4

**DOI:** 10.1017/qpb.2026.10047.pr4

**Affiliations:** Heinrich-Heine-Universität Düsseldorf, Germany

Your article “Modeling the Short-Term Response to Nitrogen that Coordinates Events in Lateral Root Initiation” has now been assessed by three expert reviewers. Please apologise for the delays in securing the reviewers´ feedback. I concur with the reviewers that your article is very interesting, potentially much relevant for the community and fits the journal spirit/aims. It has much data and of high quality and according to the state of the art. I am glad to accept the manuscript for publication provided the minor issues raised by the reviewers are tackled which will contribute to improve the article. In particular a restructuration of results/discussion, M&M; the analysis of the 3 different modules, and graphs´ quality. I do not consider any experimental work is needed but if you have any extra results you would include to strengthen some claims/improve the outcomes relevance, this is welcome.

# Editor report: Decision: Modelling the short-term response to nitrogen that coordinates events in lateral root initiation — R0/PR5

**DOI:** 10.1017/qpb.2026.10047.pr5

**Affiliations:** RDP, ENS de Lyon, France

No accompanying comment.

# Reviewer report: Review: Modelling the short-term response to nitrogen that coordinates events in lateral root initiation — R1/PR7

**DOI:** 10.1017/qpb.2026.10047.pr7

**Authors:** Thomas Blein

**Affiliations:** Universite Paris Saclay

The authors have appropriately addressed my comments by revising the text accordingly. In particular, they have corrected the identified error and provided a clear justification for the absence of a deeper analysis, citing both the limitations of the available data and the complexity of the modeling required. As they point out, a more extensive analysis would likely constitute a new study rather than simply strengthening the support for the current findings.

# Reviewer report: Review: Modelling the short-term response to nitrogen that coordinates events in lateral root initiation — R1/PR8

**DOI:** 10.1017/qpb.2026.10047.pr8

**Authors:** Francesco Sunseri

**Affiliations:** Agraria, Universita Mediterranea di Reggio Calabria, Italy

I appreciated the efforts that Authors produced in sections Results and Discussion as well as MM. Although some suggestions have not been completely checked, such as the improvement of English style, the manuscript title modification and the deletion of several citations in the results; I really appreciate the new version of the discussion. Thus, I retain this R1 version of the manuscript suitable for publication on QPB.

# Editor report: Recommendation: Modelling the short-term response to nitrogen that coordinates events in lateral root initiation — R1/PR9

**DOI:** 10.1017/qpb.2026.10047.pr9

**Affiliations:** RDP, ENS de Lyon, France

No accompanying comment.

# Editor report: Decision: Modelling the short-term response to nitrogen that coordinates events in lateral root initiation — R1/PR10

**DOI:** 10.1017/qpb.2026.10047.pr10

**Affiliations:** RDP, ENS de Lyon, France

No accompanying comment.
